# Declining HIV Prevalence in Parallel With Safer Sex Behaviors in Burkina Faso: Evidence From Surveillance and Population-Based Surveys

**DOI:** 10.9745/GHSP-D-16-00013

**Published:** 2016-06-20

**Authors:** Fati Kirakoya-Samadoulougou, Nicolas Nagot, Sekou Samadoulougou, Mamadou Sokey, Abdoulaye Guiré, Issiaka Sombié, Nicolas Meda

**Affiliations:** a Université catholique de Louvain, Brussels, Belgium; b Institut National de la Sante et de la Recherche Medicale (INSERM), UMR 1058, and Montpellier University, Montpellier, France; c Conseil National de Lutte contre le Sida (CNLS), Ouagadougou, Burkina Faso; d Organisation Ouest Africaine de la Santé, Bobo-Dioulasso, Burkina Faso; e Centre MURAZ, Bobo-Dioulasso, Burkina Faso

## Abstract

**Objective:**

To investigate trends in HIV prevalence and changes in reported sexual behaviors between 1998 and 2014 in Burkina Faso.

**Methods:**

We obtained data on HIV prevalence from antenatal care (ANC) surveillance sites (N = 9) that were consistently included in surveillance between 1998 and 2014. We also analyzed data on HIV prevalence and reported sex behaviors from 3 population-based surveys from the Demographic and Health Surveys (DHS), conducted in 1998–99, 2003, and 2010. Sex behavior indicators comprised never-married youth who have never had sex; sex with more than 1 partner; sex with a nonmarital, non-cohabiting partner; condom use at last sex with a nonmarital, non-cohabiting partner; and sex before age 15. We calculated survey-specific HIV prevalence with 95% confidence intervals (CIs) and used the chi-square test or chi-square test for trend to compare HIV prevalence across survey years and to analyze trends in reported sex behaviors.

**Results:**

HIV prevalence among pregnant women ages 15–49 decreased by 72% in urban areas, from 7.1% in 1998 to 2.0% in 2014, and by 75% in rural areas, from 2.0% in 2003 to 0.5% in 2014. HIV declined most in younger age groups, which is a good reflection of recent incidence, with declines of 55% among 15–19-year-olds, 72% among 20–24-year-olds, 40% among 25–29-year-olds, and 7% among those ≥30 years old (considering urban and rural data combined). Data reported in the DHS corroborated these declines in HIV prevalence: between 2003 and 2010, HIV prevalence dropped significantly—by 89% among girls ages 15–19, from 0.9% (95% CI, 0.2 to 1.6) to 0.1% (95% CI, 0.0 to 0.4), and by 78% among young women ages 20–24, from 1.8% (95% CI, 1.6 to 3.0) to 0.4% (95% CI, 0.0 to 0.7). During the same time period, people reported safer sex behaviors. For example, significantly higher percentages of never-married youth reported they had never had sex, lower percentages of sexually active youth reported multiple sex partners, and lower percentages of youth reported having sex before age 15. In addition, the percentage of men ages 20–49 reporting sex with a nonmarital, non-cohabiting partner declined significantly, while condom use at last sex with such a partner increased significantly among both men and women ages 15–49.

**Conclusions:**

Both ANC surveillance and population-based surveys report sharp declines in HIV prevalence in Burkina Faso between 1998 and 2014, accompanied by improvements in reported risky sex behaviors.

## Abstract (precis)

HIV prevalence among pregnant women ages 15–49 declined from 7.1% to 2.0% in urban areas between 1998 and 2014, and from 2.0% to 0.5% in rural areas between 2003 and 2014; similar declines were reported in the Demographic and Health Surveys. During the same time period, individuals reported safer sex behaviors, including delayed sexual debut and reduced number of sex partners among youth, as well as increased condom use at last sex with nonmarital partners among men and women ages 15–49.

## INTRODUCTION

HIV prevalence in Burkina Faso declined substantially over the past 10 to 15 years, from 2.2% in 2001 to 1.0% in 2012, according to a recent report from the Joint United Nations Programme on HIV/AIDS (UNAIDS).^1^ Changes in HIV prevalence result from the balance between deaths to people with HIV and incident cases of HIV in the population (along with migration patterns). Declines in HIV incidence can occur with improvements in risky sexual behaviors or antiretroviral therapy (ART) coverage and adherence among people living with HIV. Indeed, by reducing viral loads, ART reduces a person’s infectiousness through sexual transmission, and thus HIV incidence in the population.^2^ However, scale-up of ART coverage in Burkina Faso is too recent to have contributed to a decrease in prevalence among 15–24-year-olds during the last decade.

Improvements in risky sex behaviors can reduce HIV incidence.

There is considerable discussion in the literature about the declining trend in HIV prevalence among young pregnant women in sub-Saharan Africa in relation to sexual behaviors.^3^^-^^5^ In a study assessing progress in reducing HIV prevalence among young people in 30 countries around the world, Botswana, Côte d’Ivoire, Ethiopia, Kenya, Malawi, Namibia, and Zimbabwe showed a statistically significant decline of 25% or more in HIV prevalence among young antenatal care (ANC) attendees between 2000 and 2008.^5^ Moreover, in 8 countries with significant declines in HIV prevalence in either ANC surveillance surveys or national surveys, significant changes were also observed in sexual behavior in either men or women for at least 2 of 3 sexual behavior indicators (sex before age 15, multiple partners, and reported condom use among youth). Although the study could not establish causal associations between changes in sexual behavior and trends in HIV prevalence, it concluded that the observed changes were encouraging.^5^

In this article, we analyze the possible factors behind the considerable HIV decline in Burkina Faso with a focus on changes in sexual behavior.

## METHODS

We analyzed trends in HIV prevalence in Burkina Faso over a 16-year period. Data came from national ANC surveillance surveys conducted between 1998 and 2014 as well as from national surveys conducted by the Demographic and Health Surveys (DHS) program in 1998–99, 2003, and 2010. We also analyzed trends in reported sex behaviors from the DHS.

We analyzed trends in HIV prevalence in Burkina Faso from ANC surveillance and DHS surveys, along with trends in reported sex behaviors.

The HIV serosurveillance system of pregnant women was established in Burkina Faso in 1997, initially with 3 urban sites. The system evolved over time, covering 5 urban sites in 1998 and 10 urban and rural sites in 2003 (6 in urban areas and 4 in rural areas). Since 2004, 13 sites have been included in the surveillance system (the same 10 sites from 2003 plus 3 additional sites, for a total of 7 in urban areas and 6 in rural areas). The progressive increase in sentinel sites was meant to provide a roughly representative picture of levels and trends in HIV prevalence throughout the country. To avoid potential bias as a result of expanding ANC surveillance over time, only data from those urban sites that were consistently included in surveillance between 1998 and 2014 (i.e., 5 urban sites) and the rural sites included between 2003 and 2014 (i.e., 4 rural sites) were included in the analysis. Pregnant women presenting for the first time for their current pregnancy at the participating ANC sites during the survey period were enrolled in an anonymous unlinked HIV serosurvey.

We also obtained data from the DHS on HIV prevalence and reported sexual behavior. As reported elsewhere, the DHS was designed to obtain national and regional estimates of HIV prevalence and associated sociodemographic and behavioral indicators among women and men.^6^ Briefly, the DHS surveys followed a 2-stage selection process, in which a random sample of clusters from the most recent national sample frame was first selected. In the second stage, all households were listed and the final systematic random sample of households was selected. During the main fieldwork, eligible women (ages 15–49) and men (usually ages 15–59) were selected for HIV testing. In the Burkina Faso DHS, the sample was selected in 2 stages, stratified by area (urban and rural) with enumeration areas (EAs) as the first-stage sampling units and households as the second-stage sampling units.

We assessed trends in 5 key indicators related to sexual behaviors, in addition to the percentage of young people ages 15–24 years who reported having ever tested for HIV and the percentage of young people who reported knowing a formal source of condoms. The behavioral indicators were:

- The percentage of never-married young women and men ages 15–24 who have **never had sex**
- The percentage of young people (ages 15–24) who have had **sex with more than 1 partner** in the last 12 months among all young people who have been sexually active in the last 12 months
- The percentage of respondents who have had **sex with a nonmarital, non-cohabiting partner** in the last 12 months among all respondents reporting sexual activity in the last 12 months
- The percentage of respondents who reported **using a condom the last time they had sex with a nonmarital, non-cohabiting partner** among those who have had sex with such a partner in the last 12 months
- The percentage of young women and men ages 15–24 who have had **sex before age 15**

To describe HIV prevalence trends by data source, we calculated survey-specific HIV prevalence with 95% binomial confidence intervals (CIs). To determine the relative proportional change in prevalence across survey year, the difference between estimates from the earlier and later rounds was divided by the earlier estimate. When comparing HIV prevalence across survey years, we used the chi-square test or the chi-square test for trend. Trends in reported sexual risk behavior were analyzed using the same tests.

## RESULTS

### Trends in HIV Prevalence

**FIGURE 1:**
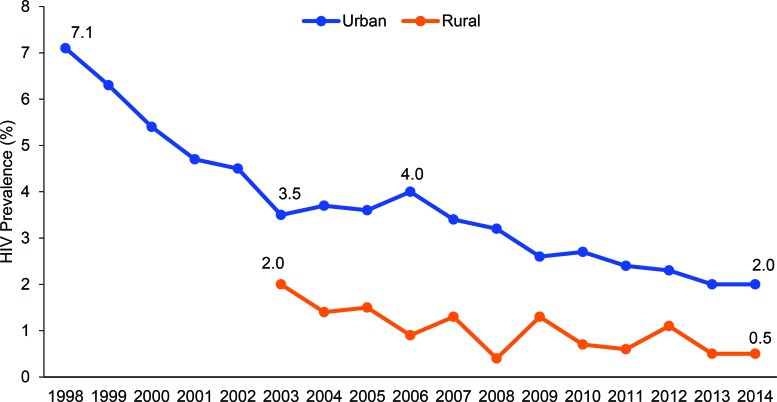
Trends in HIV Prevalence Among Pregnant Women Ages 15–49 Surveyed at Urban and Rural Antenatal Sentinel Surveillance Sites, Burkina Faso, 1998 to 2014

The number of pregnant women ages 15–49 years included in the ANC surveillance surveys varied from 2,010 in 1998 (from urban areas only) to 3,129 in 2014 (from urban and rural areas), with a maximum of 3,276 in 2008 (urban and rural). Between 1998 and 2003, HIV prevalence declined among 15–49-year-old pregnant women in urban areas (data not collected in rural areas) from 7.1% to 3.5%, a decline of 51% (Figure 1). While prevalence increased somewhat in urban areas between 2003 and 2006, from 3.5% to 4.0%, by 2007, prevalence had begun to decline again and continued to drop—to 2.0% in 2014. HIV prevalence among pregnant women in rural areas followed similar trends, falling from 2.0% in 2003 to 0.5% in 2014 (Figure 1).

Between 1998 and 2014, HIV prevalence declined among pregnant women ages 15–49 in both urban and rural areas.

**FIGURE 2:**
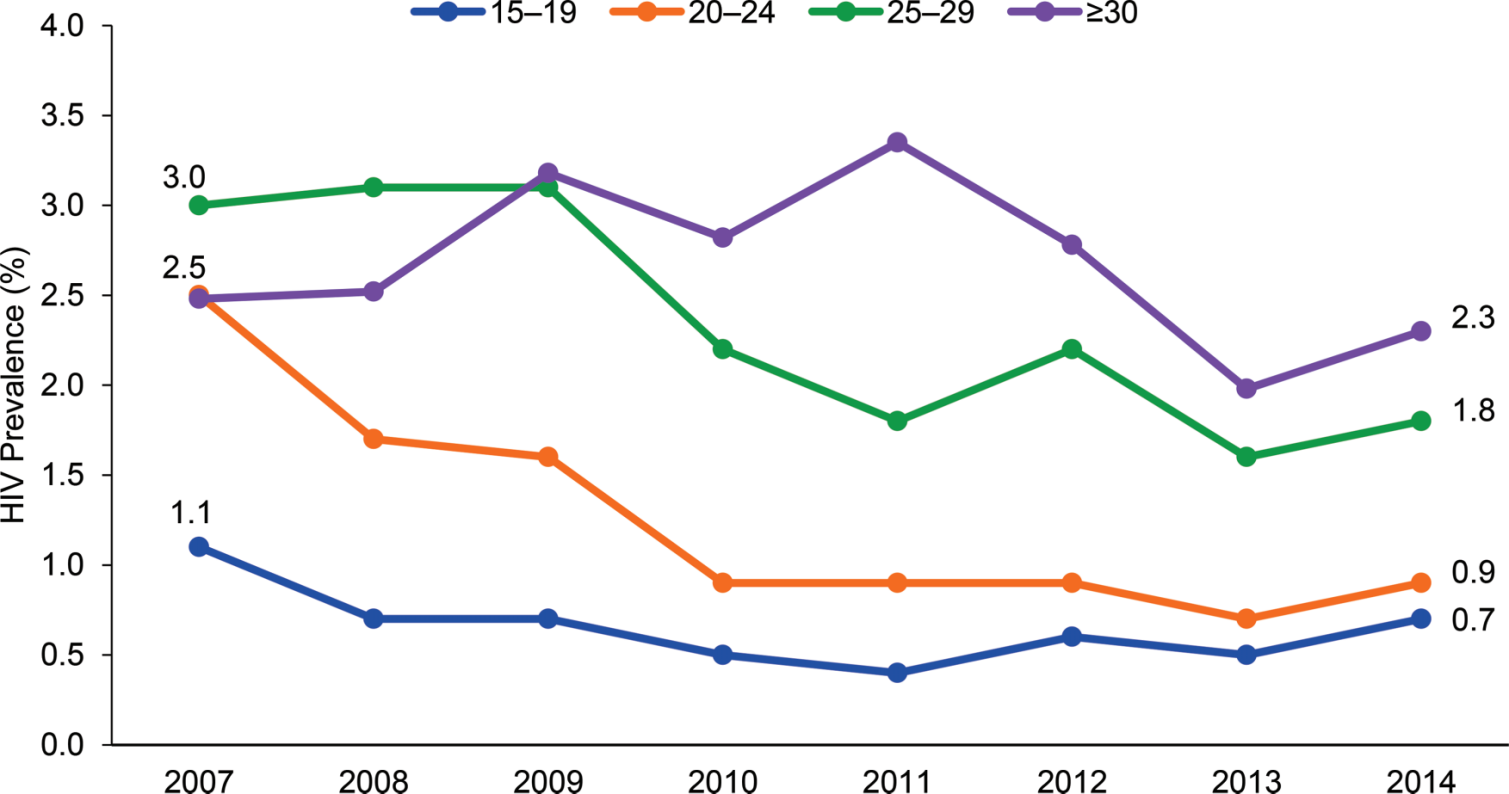
Trends in HIV Prevalence Among Pregnant Women Ages 15–49 Surveyed at Urban and Rural Antenatal Sentinel Surveillance Sites, by Age Group, Burkina Faso, 2007 to 2014

Age-specific data were available between 2007 and 2014. These data (urban and rural areas combined) indicate HIV prevalence declined most in younger age groups with declines of 55% among 15–19-year-olds, 72% among 20–24-year-olds, 40% among 25–29-year-olds, and 7% among those ≥30 years old (Figure 2).

HIV prevalence declined most in younger age groups.

**FIGURE 3:**
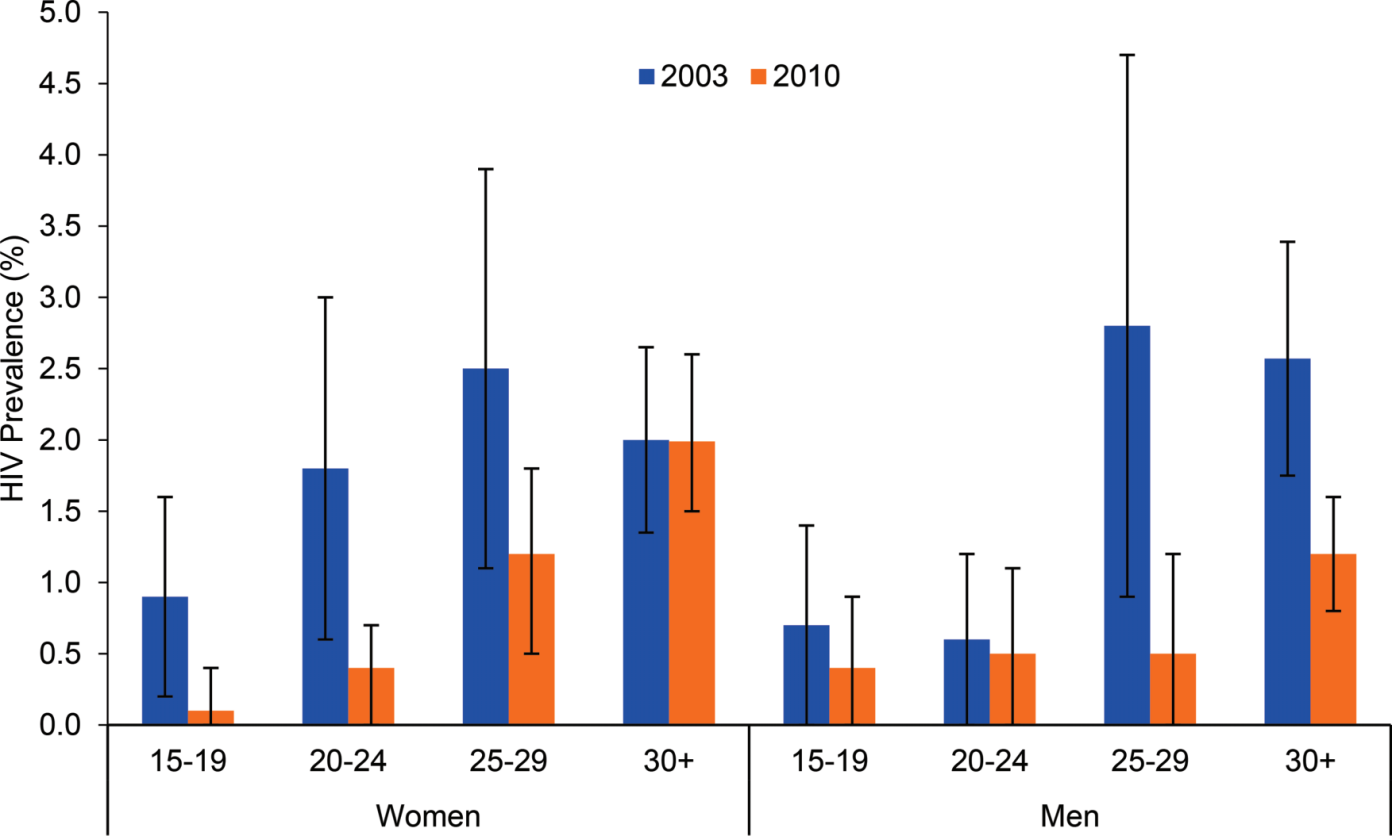
Trends in HIV Prevalence (With 95% Confidence Intervals) Among Women and Men Surveyed in the Demographic and Health Surveys, by Age Group, Burkina Faso, 2003 and 2010

The decline in HIV prevalence among pregnant women in the ANC surveillance surveys was consistent with HIV prevalence data collected by the DHS. Between the 2003 and 2010 DHS, HIV prevalence among girls ages 15–19 dropped by 89% (*P* = .04), from 0.9% (95% CI, 0.2 to 1.6) to 0.1% (95% CI, 0.0 to 0.4) (Figure 3). Among young women ages 20–24, prevalence declined by 78% (*P* = .001) over the same time period, from 1.8% (95% CI, 1.6 to 3.0) to 0.4% (95% CI, 0.0 to 0.7), and among women ages 25–29, it dropped by 52%, from 2.5% (95% CI, 1.1 to 3.8) to 1.2% (95% CI, 0.5 to 1.8).

Among men, the decline in HIV prevalence was much less marked among the younger age groups but more substantial among the older groups. For example, among boys ages 15–19, HIV prevalence decreased by 43% between 2003 and 2010, from 0.7% (95% CI, 0.0 to 1.5) to 0.4% (95% CI, 0.0 to 0.9) , whereas for men ages 25–29 HIV prevalence declined significantly by 82% (*P* = .001), from 2.8% (95% CI, 0.9 to 4.7) to 0.5% (95% CI, 0.0 to 1.2) (Figure 3).

### Trends in Sexual Behaviors

**FIGURE 4:**
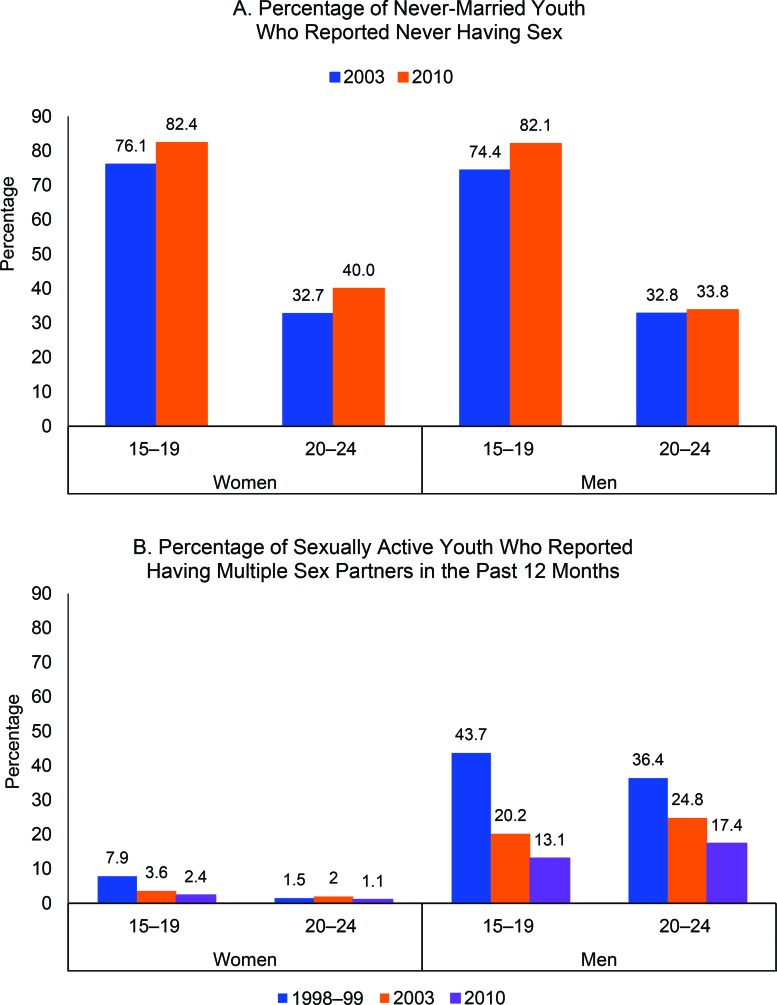
Reported Sex Behaviors of Youth Ages 15–24, by Sex and Age Group, Burkina Faso, 2003 and 2010

The percentage of never-married adolescents ages 15–19 who reported they had **never had sex** increased significantly between 2003 and 2010 among both girls and boys: for girls, from 76.1% to 82.4% (*P*<.001); for boys, from 74.4% to 82.1% (*P*<.001) (Figure 4A). The percentage of never-married young women ages 20–24 who reported never having had sex also increased significantly over the same time period, from 32.7% to 40.0% (*P*<.001). Among never-married young men ages 20–24, the increase was smaller and not statistically significant: 32.8% in 2003 to 33.8% in 2010 (*P* = .68).

The percentage of never-married adolescents who reported they had never had sex increased significantly between 2003 and 2010.

The percentage of sexually active girls ages 15–19 who reported having **multiple partners** in the last year fell from 7.9% to 2.4% (*P*<.001) between 1998–99 and 2010, whereas a smaller decline was observed among sexually active young women ages 20–24 (1.5% in 1998–99 to 1.1% in 2010; *P* = .04) (Figure 4B). Substantial declines were observed among sexually active males ages 15–19 and 20–24 during the same time period: from 43.7% to 13.1% (*P*<.001) and from 36.4% to 17.4% (*P*<.001), respectively.

The percentage of youth reporting multiple sex partners declined significantly between 1998 and 2010.

**FIGURE 5:**
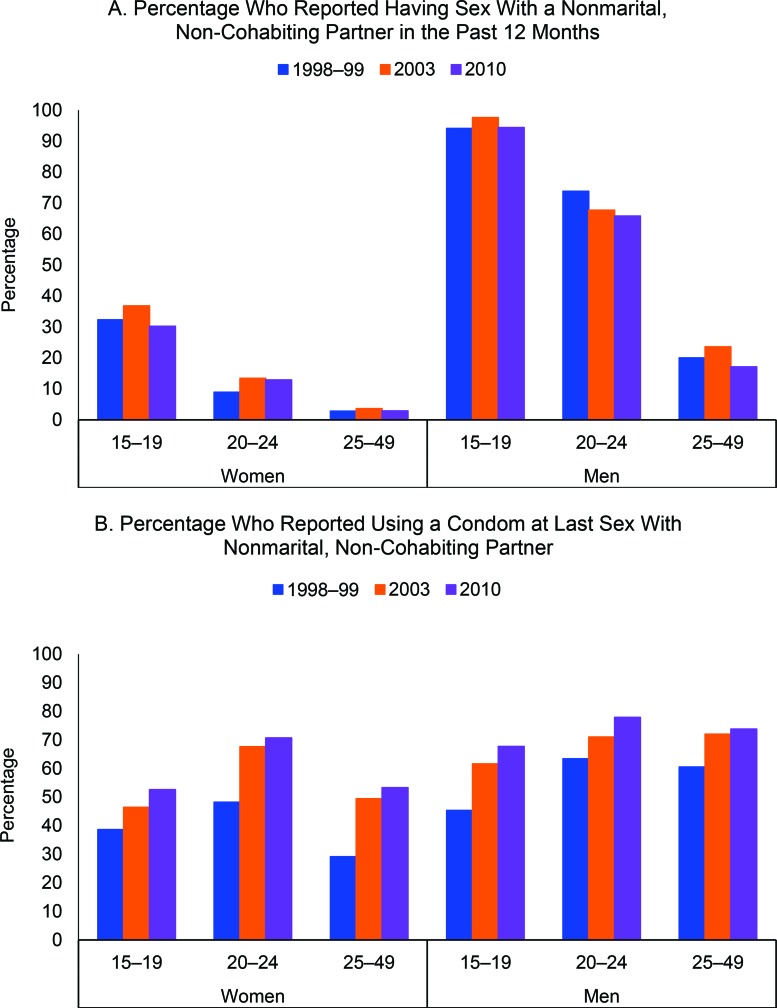
Reported Sex Behaviors Among Sexually Active Women and Men Ages 15–49, by Sex and Age Group, Burkina Faso, 1998–99 to 2010

The percentage of sexually active women ages 15–49 reporting engaging in **sex with a nonmarital, non-cohabiting partner** in the last year has always been quite low and has remained stable during the study period (8.3% in 1998–99 to 7.9% in 2010). The percentage of sexually active boys ages 15–19 who reported engaging in sex with a nonmarital, non-cohabiting partner in the last 12 months also remained unchanged (94.1% in 1998–99 to 94.2% in 2010), whereas the percentages declined significantly from 73.8% to 65.8% (*P* = .009) among young men ages 20–24 and from 20.0% to 17.1% (*P*<.001) among older men ages 25–49 (Figure 5A).

The percentage of sexually active men ages 20–24 and 25–49 who reported having sex with a nonmarital partner declined significantly between 1998 and 2010, while it remained stable and low among women.

The percentage of women and men ages 15–49 who have had sex with a nonmarital, non-cohabiting partner in the last 12 months who said they used a **condom the last time they had sex** with such a partner increased significantly between 1998–99 and 2010, from 38.6% to 59.0% (*P*<.001) among women and from 57.4% to 73.9% among men (*P*<.001). This percentage increased significantly among girls ages 15–19, from 38.6% in 1998–99 to 52.6% in 2010 (*P*<.001), as well as among boys ages 15–19, from 45.3% to 67.7% (*P*<.001). Similar trends were also evident among men and women in older age groups (Figure 5B).

The percentage of men and women ages 15–49 who reported using a condom at last sex with a nonmarital partner increased significantly between 1998 and 2010.

Among both young women and men ages 15–24, the percentage having **sex before age 15** declined between 1998–99 and 2010: from 11.2% to 9.3% (*P* = .02) among young women and from 7.6% to 1.9% (*P*<.001) among young men.

The percentage of youth who reported having sex before age 15 declined significantly between 1998 and 2010.

**FIGURE 6:**
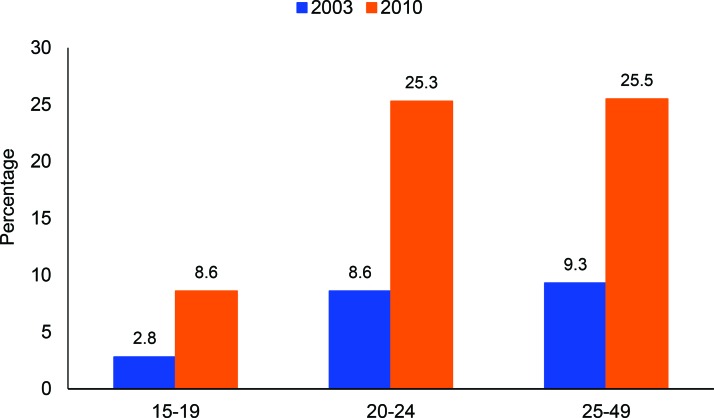
Percentage of Men Who Have Ever Received an HIV Test, by Age Group, Burkina Faso, 2003 and 2010

**HIV testing** rates among men increased significantly between 2003 and 2010, increasing most in younger age groups—by 67.4% among 15–19-year-olds (*P*<.001) and 66.0% among 20–24-year-olds (*P*<.001), compared with 63.5% among 25–49-year-olds (*P*<.001) (Figure 6). Overall, 27.3% (95% CI, 25.5 to 29.2) of women ages 15–24 were ever tested for HIV in 2010. Unfortunately, HIV testing among women was not collected in 2003, so analysis of trends is not possible.

The percentage of adolescent girls and boys ages 15–19 who reported **knowing a formal source of condoms** increased from 44.3% to 73.1% (*P*<.001) and from 63.1% to 85.0% (*P*<.001), respectively, between 2003 and 2010. The percentage among young women and men ages 20–24 increased from 49.6% to 79.6% (*P*<.001) and from 83.3% to 96.9% (*P*<.001), respectively, between 2003 and 2010.

## DISCUSSION

We analyzed and assessed, for the first time, all data available on trends in HIV prevalence in Burkina Faso from the onset of the epidemic. These data provide a consistent picture of a decline in HIV prevalence among pregnant women ages 15–49 starting in 1998 and continuing up to 2003, from 7.1% to 3.5%, followed by further decline after 2007 to 2.0% in 2014. National DHS data also indicate a reduction of HIV prevalence in the country. The declining trend in HIV prevalence among pregnant women, particularly those ages 15–19 described here, is consistent with data from several other sub-Saharan African countries during a similar time period.^3^^-^^5^^,^^7^^,^^8^

We also presented the first comparisons of data on trends in sexual behavior of comparable populations over time since the onset of the HIV epidemic. The percentage of never-married young women and men ages 15–24 who have never had sex increased substantially during precisely the same time period as when HIV prevalence dropped. Furthermore, the percentage of young individuals reporting having multiple partners decreased, particularly among males, while reported condom use at last sex with non-regular partners increased. Taken together, these trends suggest that the reduction in HIV prevalence was, at least in part, due to a reduction in HIV incidence but also to a strong decline in HIV prevalence among older men (who are usually partners of younger women). Indeed, HIV prevalence among young people ages 15–24 can provide useful indications of trends in HIV incidence, and behavior change among older people, particularly men, could cause reductions in prevalence among young people. The results of these nationwide surveys are consistent with cross-sectional surveys of HIV prevalence and risky behaviors in other countries, all of which demonstrate that the most common behavioral changes involved delays in sexual initiation,^9^ reductions in sex outside of marriage,^3^^,^^10^ declines in number of sex partners, and increases in condom use.^4^^,^^11^^,^^12^

The temporal order of these changes in behavior and declines in HIV prevalence after 2007 is supported by programmatic efforts occurring during that time. From 2006 to 2010, Burkina Faso established an HIV strategic framework with central coordination. The number of sites for the prevention of mother-to-child transmission of HIV (PMTCT) rose to 780 in 2007, 3 times higher than in 2006, and climbed to 1,226 in 2010. From 2007 onwards, awareness campaigns on female and male condom use were active in all sectors (e.g., schools, health centers, and public places). Moreover, the number of health facilities with a counseling center and a voluntary testing center tripled between 2006 and 2007, from 284 to 837, continuing to increase to 1,305 in 2010.^13^

The decline in HIV prevalence in the Burkina Faso DHS was much higher among young women than young men. In 2010, HIV prevalence among young men was as high, or higher, than among women, which is very unusual. In Africa as a whole, women ages 15–24 are infected with HIV at rates 2.5 times that of young men.^14^ The gap in HIV decline between men and women may be linked to the scale-up of PMTCT programs. Access to PMTCT can lead to enhanced preventive behavior such as condom use or choice of partners. HIV infection among young women may also be influenced by the risk behavior or infectivity of their sexual partners, who are usually a few or more years older than them.^15^^-^^17^ In our analysis, we observed a substantial decline in HIV prevalence among older men (≥25 years) along with a lower percentage of adults (≥25 years) reporting 2 or more sexual partners in the last year and an increase in reported condom use. The higher prevalence in young men than young women in Burkina Faso may also reflect to some extent HIV transmission among men who have sex with men. Indeed, Burkina Faso is a country that has very high rates of male circumcision (88.7% in 2010 and 90.4% in 2003 among males ages 15–49); the relatively low prevalence and incidence of HIV in the country is generally credited with the high rates of male circumcision. Accordingly, the transmission dynamic of HIV is probably more a function of most-at-risk people such as sex workers and men who have sex with men. Changes in sexual behaviors have also been accompanied by a reduction of HIV incidence among female sex workers in West Africa. A recent study among female sex workers ages 18–25 years in Burkina Faso showed that combining peer-based prevention and care within the same setting markedly reduced HIV incidence through reduced risky behaviors.^18^ This reduction in HIV cases among female sex workers likely has an impact on HIV prevalence in the general population since more than half of new HIV cases in sub-Saharan Africa are linked with female sex worker contacts.^19^

### Limitations

Although HIV decline in Burkina Faso was paralleled with safer sexual behaviors, these findings have limitations. Data collected on sexual behaviors over time may be subject to social desirability bias, as prevention programs can change the social norms regarding sexual behavior.^20^ In addition, although the HIV prevalence data from the ANC surveillance surveys confirm the trends observed in the DHS data, pregnant women are not representative of the general population. In general, estimates based on pregnant women tend to overestimate HIV prevalence among all women at young ages, due to selection for sexual activity,^21^ as we observed in the current study. The Burkina Faso DHS sample may also be biased due to differential non-response in the survey and/or exclusion of most-at-risk persons. Finally, the current analysis cannot establish a causal association between changes in sexual behavior and trends in HIV prevalence. Although HIV incidence was certainly reduced among young people ages 15–25, we cannot exclude the potential role of mortality in this age group: individuals who get infected with HIV at 19–20 years can die of the infection by 25 years if they do not receive treatment.

## CONCLUSION

Findings from the current study are consistent with previous work indicating that observed declines in HIV prevalence or incidence were coincident with reductions in sexual risk behavior in countries with generalized HIV epidemics.^12^ The broad range of HIV education and prevention programs in Burkina Faso that made use of national media along with school- and workplace-based activities and other interpersonal communication interventions may have contributed to the declines in HIV prevalence by helping to reduce risky sex behaviors among youth. Results were particularly encouraging among young women; stronger interventions targeting young men are needed to reinforce the control of the HIV epidemic in Burkina Faso.

Declines in HIV prevalence were coincident with reductions in sexual risk behavior in Burkina Faso.

## Notes

**Peer Reviewed**
